# Copy Number Variation That Influences the Ionizing Radiation Sensitivity of Oral Squamous Cell Carcinoma

**DOI:** 10.3390/cells12202425

**Published:** 2023-10-10

**Authors:** Tadahide Izumi, Piotr Rychahou, Li Chen, Molly H. Smith, Joseph Valentino

**Affiliations:** 1 Department of Toxicology and Cancer Biology, University of Kentucky, Lexington, KY 40536, USA; 2 Markey Cancer Center, University of Kentucky, Lexington, KY 40536, USA; 3 Department of Surgery, University of Kentucky, Lexington, KY 40536, USA; 4 Internal Medicine, University of Kentucky, Lexington, KY 40536, USA; 5 Oral Pathology, University of Kentucky, Lexington, KY 40536, USA; 6 Pathology and Cytology Laboratory, University of Kentucky, Lexington, KY 40506, USA; 7 Department of Otorhinolaryngology, University of Kentucky, Lexington, KY 40536, USA

**Keywords:** radiation resistance, oral squamous cell carcinoma, copy number variation, genome instability, DNA repair

## Abstract

Genome instability in cancer cells causes not only point mutations but also structural variations of the genome, including copy number variations (CNVs). It has recently been proposed that CNVs arise in cancer to adapt to a given microenvironment to survive. However, how CNV influences cellular resistance against ionizing radiation remains unknown. PRMT5 (protein arginine methyltransferase 5) and APE1 (apurinic/apyrimidinic endonuclease 1), which enhance repair of DNA double-strand breaks and oxidative DNA damage, are closely localized in the chromosome 14 of the human genome. In this study, the genomics data for the PRMT5 and APE1 genes, including their expression, CNVs, and clinical outcomes, were analyzed using TCGA’s data set for oral squamous cell carcinoma patients. The two genes were found to share almost identical CNV values among cancer tissues from oral squamous cell carcinoma (OSCC) patients. Levels of expression of PRMT5 and APE1 in OSCC tissues are highly correlated in cancer but not in normal tissues, suggesting that regulation of PRMT5 and APE1 were overridden by the extent of CNV in the PRMT5-APE1 genome region. High expression levels of PRMT5 and APE1 were both associated with poor survival outcomes after radiation therapy. Simultaneous down-regulation of PRMT5 and APE1 synergistically hampered DNA double-strand break repair and sensitized OSCC cell lines to X-ray irradiation in vitro and in vivo. These results suggest that the extent of CNV in a particular genome region significantly influence the radiation resistance of cancer cells. Profiling CNV in the PRMT5-APE1 genome region may help us to understand the mechanism of the acquired radioresistance of tumor cells, and raises the possibility that simultaneous inhibition of PRMT5 and APE1 may increase the efficacy of radiation therapy.

## 1. Introduction

Oral squamous cell carcinoma (OSCC) is the sixth most common cancer worldwide. A recent estimation of the annual incidence of cancer of the oral cavity and pharynx (Tongue, mouth, pharynx and other oral cavity) in the US is about 54,000, and the estimated mortality is 11,580 (females and males combined) [1]. These numbers have increased since 2016 (48,330 and 9570, respectively) [2]. More than 60% of OSCC patients who undergo radiotherapy often find tumors recur [3,4,5,6], with increased resistance to ionizing radiation (IR) and a significantly lower survival rate [7]. However, the mechanism of the acquired IR resistance of OSCC remains elusive, despite the immense research on cellular defense mechanisms including DNA repair functions [8,9,10]. IR generates DNA double-strand breaks (DSBs) both directly or indirectly through accumulation of DNA single-strand breaks (SSBs) and oxidative DNA damage [11]. Cells need to repair clusters of damage that are mixtures of DSB and oxidative damage [12,13,14,15,16,17]. This implies that the mechanism of acquired IR resistance involves multiple DNA repair pathways. However, whether and how cancer cells increase these activities simultaneously has not been studied mechanistically in relation to cancer genetics. 

Cells possess two main DSB repair (DSBR) pathways, i.e., DSBR via homologous recombination (HR) and non-homologous end joining (NHEJ). Enhancement of either DSBR pathway may confer resistance to radiation. Owens et al. recently reported that protein arginine methyltransferase 5 (PRMT5) is a crucial activator of both HR and NHEJ DSBR [18]. Accordingly, PRMT5 methylates an arginine residue of histone 4 (H4R3), and activates promoters of HR genes (RAD51, RAD51D, RAD51AP, BRCA1, BRCA2) and NHEJ genes (NHEJ1, DNAPKcs, Ku80, XRCC4) [18]. Down-regulation of PRMT5 sensitized HeLa cells and prostate cancer cells to radiation [18,19], underscoring its crucial role in cellular defense against IR.

On the other hand, oxidative DNA damage is repaired by the DNA base excision repair (BER) pathway, in which apurinic/apyrimidinic (AP) endonuclease 1 (APE1) converts AP sites and 3′-blocking SSBs to 3′-OH termini, which are absolutely required for DNA synthesis [20]. APE1 was reported to be the rate-limiting factor in the entire BER reaction for the repair of oxidative DNA damage [21].

It was separately shown that down-regulation of either PRMT5 or APE1 sensitized cells to IR [18,19,22]. Increases in both proteins in cancer cells were independently reported [23,24]. Because PRMT5 and APE1 functions in the different DNA repair pathways, simultaneous up- or down-regulation of PRMT5 and APE1 may significantly affect the IR susceptibility of cancer cells. However, no studies have examined the possibility of correlation between the expression of PRMT5 and APE1. Here, we report that the levels of their expressions are highly correlated, specifically in cancer tissues. We provide evidence that the high expressional correlation between PRMT5 and APE1 is mainly due to the close localization of the PRMT5 and APE1 genes in the chromosome 14 of the human genome (the physical map based on GRCh38/hg38 are chr14:22,920,525–22,929,408 (PRMT5) and chr14:20,455,191–20,457,772 (APEX1), https://www.genecards.org, accessed on 2 September 2023). Simultaneous down-regulation of PRMT5 and APE1 synergistically sensitized OSCC cell lines in vitro and in vivo. Shih et al. recently identified that cancer cells take advantage of particular copy number variations (CNVs) in their growth environments [25]. We determined that the extent of the CNVs of these two genes is almost identical among the tumor tissues, and present the indication that this reflects a high correlation in their expression.

## 2. Materials and Methods

### 2.1. Clinical and RNAseq Data of TCGA

RNAseq and clinical records of OSCC patients were obtained from TCGA (https://portal.gdc.cancer.gov/legacy-archive/search/f, accessed on 2 September 2023). They include RNAseq, SNP array, and clinical records from 522 patients. De-identified clinical records in json format were used to extract cross-referenced numbers (patient_id) that were linked to the genomics data, pathological cancer stages, information regarding radiation treatment (labeled “radiation_treatment” and “radiation_dose”). Dates of death and last dates for follow-ups were combined to build survival records.

TCGA RNAseq data for the OSCC tissues of the 522 patients and 43 matched normal tissues were also analyzed. The RNAseq data (normalized gene intensities) were consolidated to a table containing all RNA expression intensities and the patient_id numbers. There are genes of which expression was almost undetectable, resulting in 0 RNAseq intensities for most of the specimens. Such genes with low RNAseq intensities (0 intensities in 10% or more of the specimens) were ignored and not analyzed in this study. Through this process, data of 15,858 genes were used for further analyses. Patients whose tumors were pathologically diagnosed as T4 stage and who underwent radiation therapy were selected and analyzed for this study. Relevant transcription table and clinical records are provided as supplemental files.

### 2.2. Associating Gene Coordinate to TCGA RNAseq Data

RNAseq data were linked to the survival data of the corresponding individuals. For each gene, two RNAseq (and the survival) datasets, QTR1 and QTR4, were grouped based on their expression levels. QTR1 (the lower quartile) consists of those of the minimum to the first quartile, and QTR4 (the upper quartile) contains data from the third quartile to the maximum in terms of gene expression intensities. Then, difference in the survival between the QTR1 and QTR4 groups was examined using a Kaplan–Meier estimator. *p*-values were calculated with a log-rank test using the survdiff function in R’s survival package [26].

### 2.3. Genomic Copy Number Assessment of TCGA SNP Array

The transcription start sites of genes were obtained from the Entrez database based on GRCh38, and were used as genetic coordinates. Masked copy number segment values were determined using data from Affymetrix SNP array 6.0 (https://docs.gdc.cancer.gov/Data/Bioinformatics_Pipelines/CNV_Pipeline/, accessed on 5 October 2023), for 522 OSCC patients versus their normal controls. Based on a manifest file that included a file name and patient id as well as specimen tags, the copy number data files were separated to tumor- and normal- (obtained from blood) segment copy files. Each file, consisting of lines starting and ending with GRCh38 genomic coordinates, number of probes, and the segment mean values, were consolidated into location-specific mean CNV values at the transcription starting positions of the analyzed genes, including PRMT5 and APE1.

### 2.4. Cell Lines

An hypopharynx squamous cell carcinoma cell line FaDu [27] was purchased from the American Tissue Culture Collection (ATCC). Genomic DNA from these cell lines were analyzed with short tandem repeat probes for authentication (LabCorp, Burlington, NC, USA). Cells were cultured in a 5% CO_2_ incubator in Dullbeco’s modified Eagle’s medium (DMEM, Gibco/Thermofisher, Waltham, MA, USA) supplemented with 10% fetal bovine serum (Geminibio, West Sacramento, CA, USA), 20 mM L-Glutamine (Gibco/Thermofisher), and penicillin/streptomycin mix (100 units/mL, Gibco/Thermofisher).

### 2.5. Transient RNA Interference with siRNA

Transient down-regulation of APE1 and PRMT5 expression were achieved with electroporation of specific siRNA oligonucleotides. Plates containing 80–90% confluent FaDu cells were washed with phosphate-buffered saline (PBS) twice and trypsinized, and suspended in the complete medium. Adjusted to 1 × 10^7^ cells/mL, 100–200 µL cell suspension was mixed with siRNA oligonucleotides at the final concentration of 200 nM. The siRNA were purchased from Ambion/Thermofisher (Waltham, MA, USA) (APE1, s1445) and IDT (PRMT5, hs.RiPRMT5.13.1). The mixtures were then transferred into 0.4 cm EBC electroporation cuvettes, and electroporated using ECM830 (BTX) at 900 V, 120 µF, with 2 pulses of 0.1 s interval, which induced no detectable cytotoxicity to FaDu cells. Cells were then left at room temperature for 15 min, and then plated in dishes containing the complete medium. Cells were then incubated for 48 h, and then replated for a survival assay or protein analysis next day (total 72 h incubation).

### 2.6. Stable Cell Lines with Lentiviral shRNA Vectors

Lentiviral vectors from Addgene were used to generate FaDu derivatives stably expressing shRNA specific to PRMT5 and APE1 transcripts. The vectors were pLKO.1-puro (id 8453, Addgene, Watertown, MA, USA), pLKO.1-blast (26655). Oligonucleotides for shRNA were inserted into *Age*I-*Eco*RI sites or *Nhe*I-*Eco*RI sites, respectively, in the vectors above. HEK293Ta cells were transfected with the shRNA vectors with helper DNA (pMD2.G and psPAX2), using ViaFect transfection reagent (Promega, Madison, WI, USA). Media from these transfection dishes were then used to infect the cells. Selections of stable derivatives were made with appropriate antibiotics: 1 µg/mL puromycin (Invivogen/Thermofisher, Waltham, MA, USA) and 5 µg/mL blasticidine (Invivogen/Thermofisher).

### 2.7. Immunoblot Assay

Cells were washed in PBS twice, and then lysed in a lysis buffer (20 mM Tris-Cl pH 7.4, 150 mM NaCl, 1 mM EDTA, 1% Triton-X, 0.1% SDS, 1 mM DTT) with sonication for 10 s. Protein concentrations were determined using Bradford reagent (Bio-Rad, Hercules, CA, USA), and adjusted to an equal concentration among the sample groups. The cell lysates were run in 4–20% gradient SDS/PAGE (Bio-Rad). The proteins were then electroblotted on PVDF membranes (Bio-Rad). Immunoblot assays were then carried out to detect APE1 and PRMT5 using the method previously described [28]. The primary antibodies for APE1 (sc-55498) and PRMT5 (sc-376937) were purchased from SCBT (Santa Cruz Biotechnology, Dallas, TX, USA). 

### 2.8. Comet Assay

The comet assay [29] reagents, including low melting agarose, lysis buffer, and slides, were included in the comet assay kit (4250-050-K) purchased from R&D systems (Minneapolis, MN, USA). FaDu cells and their shRNA knockdown (kd) derivatives were plated on to 6 cm dishes one day before X-ray irradiation. Cells were spread on the Petri dishes, so that the cell density (confluency) was about 70% on the plate, to prepare an appropriate density of the cell/gel mixture at a later stage. Cells were irradiated with 4.5 Gy X-ray using a 225 kV orthovoltage X-ray irradiator (XRAD-225XL, PXi, Branford, CO, USA). The doses were calculated with consideration of the backscattering effect [30] by the X-ray service core at the Department of Toxicology and Cancer Biology of the University of Kentucky [30,31]. The cells were then incubated at 37 °C for 30 min, 6 h, and 24 h, and were harvested at the same time. Cells were washed with PBS twice and trypsinized for 2 min at room temperature, and immediately mixed in low-melting-point agarose at 37 °C and poured on to comet assay slides. After 10 min at 4 °C, the cells were lysed in the lysis buffer at 4 °C for 1 h, followed by incubation in Tris/Borate/EDTA (TBE) buffer (Bio-Rad) at 4 °C for 30 min. Gel electrophoresis was then carried out in the TBE buffer at 4 °C at 1 V/cm for 30 min. The DNA inside of gels was then immobilized by incubating the slides in 1 M NH_4_-acetate/ethanol solution for 30 min at room temperature, as described in the vendor’s manual, followed by incubation in 70% ethanol for 30 min. The gels in the slides were then dried, and stained in a SYBRgold solution (original stock ×10,000-dilution) in TE buffer. After rinsing in H_2_O briefly, the slides were completely dried and were covered with an anti-fade solution (S3023, DAKO, Glostrup, Denmark). The comet signals were photographed using a Nikon TE microscope (Melville, NY, USA) in the tag image file format (tiff) at 1024 × 1024 resolution, and then analyzed using Comet Assay software (R&D Systems). The parameter of tail moment (the product of distance and intensity integrated over the tail length to assess the severity of damage) is defined using this software.

### 2.9. X-ray Survival Assay

A colony formation assay was performed to determine survival fractions for FaDu cells. One day before X-ray exposure, cells were spread on 6-well culture dishes at 1000 cells per well for FaDu. Cells were then incubated for 24 h. X-ray exposure was carried out using a 225 kV orthovoltage X-ray irradiator, as described above. Cells were then incubated in a CO_2_ incubator for 9–10 days. After colony formation, cells were fixed and stained in a crystal violet solution [32]. Having been washed in water and dried, colonies were counted under a Zeiss stereo microscope (Stemi DV4 Hebron, Kentucky, KY, USA). Dead cells that did not divide but became enlarged and left recognizable crystal violet stains were excluded from counting after microscopic observations.

### 2.10. Determination of Copy Number Variation of OSCC Tissues

The copy number variations in OSCC tissues were determined with a Nanostring CNV cassette (Seattle, WA, USA) customized to detect the APE1, PRMT5, and other genes inside of the APE1-PRMT5 genome region, using a method described recently [33]. Briefly, formalin-fixed, paraffin-embedded (FFPE) oral cancer tissues preserved at the oral pathology laboratory of the University of Kentucky were pathologically reviewed to determine squamous cell carcinoma regions. The FFPE tissues had been de-identified, which did not involve any investigators participating in this study. The University of Kentucky Institutional Review Board determined this study to be IRB-exempt. Genomic DNA from these regions was extracted using a Pinpoint Slide DNA Isolation kit (Zymo research, D3001, Irvine, CA, USA). Normal human genomic DNA was used as the control genome DNA, which provides the reference values for the diploid DNA. A total of 320 ng of the genomic DNA was digested with *Alu*I restriction endonuclease, which was provided to produce DNA fragments of 200–300 bp size on average, according to the manufacturer’s protocol. The digested DNA was then analyzed in nCounter Sprint using the Oncogenomics core facility of the Markey Cancer Center. 

### 2.11. Tumor Growth Assay in Mouse Xenograft

The animal protocol was approved by the Institutional Animal Care and Use Committee (IACUC) at the University of Kentucky. Tumor growth using athymic mice was carried out using FaDu and its derivatives. FaDu derivatives expressing single shRNA (sh-control, sh-APE1, sh-PRMT5) and both shRNA (shPRMT5 and shAPE1) were established to down-regulate these factors stably, using the lentiviral vectors described as above. The cells (1.0 × 10^6^) were injected subcutaneously into the mouse flank on a warming platform and under anesthesia with oxygenated isoflurane. When the tumors reached a size of approximately 200 mm^3^, the mice were anesthetized with ketamine (100 mg/Kg)/xylazine (10 mg/Kg) and were irradiated with a total of 12 Gy (4 Gy × 3 day) at the tumor sites in the X-ray Service Center at the Department of Toxicology and Cancer Biology of the University of Kentucky, using PXi 225XL-RAD [30,31,34]. X-ray irradiation was controlled with an adjustable collimator to focus on the tumor site specifically. 

### 2.12. Statistical Methods

All in vitro experiments were repeated at least three times. Each survival treatment was triplicated, and the reproducibility of the results was assessed by repeating each experiment at least three times. To statistically evaluate the comet assay results, tail moment values obtained using Comet Assay software (R&D Systems) were compared among the cell lines with Welch’s *t*-test, and adjusted using the Bonferroni method. To evaluate the X-ray sensitivity of the tumors in the mouse xenograft, growth in tumor size was measured twice a week with the formula 6/π • (wider length)•(shorter length)/2. Tumor growths among groups were compared for their longitudinal growth difference based on a linear mixed model with Bonferroni adjustment using the R-package TumGrowth [35]. For CNV evaluation of FFPE OSCC tissues based on NanoString, the relative intensities of three independent NanoString code sets per each gene were normalized with corresponding control values obtained with human diploid standard genome DNA (Promega, G147A), and were used to obtain mean values of CNV for each gene in the 14q11.2 region. The *p*-values were adjusted with Holm’s method for multiple comparisons, as shown in Table S1.

## 3. Results

### 3.1. Overall Survival Analysis

There were 522 OSCC cases deposited in TCGA, with gene expression profiles and clinical records. Based on information regarding radiation dosage in the clinical record “rad:radiations”, there were 308 cases with radiation treatment. There were 97 Stage 4 cases (locally advanced tumors) that were treated with radiation. However, two individuals in this group underwent radiation in unusually low doses (0.5 and 0.6 Gy). These two cases were considered outliers and were excluded from this study. The mean and median doses of X-ray in the remaining 95 cases were 60 and 58.36 Gy, respectively. The mean dosage was almost identical to those of all stages, but with a lower s.d. than those of the T4 stage (standard deviation: 11.3 for T4 versus 15.2 for other stages), indicating that the radiation exposure method is more consistent among T4-stage patients than those of all stages. Additionally, the “radiation_therapy” tag in the clinical sheet listed 53 cases with “YES”, which were included in the 95 cases described above. The mean dosage of the 53 cases was 60.23 (s.d. = 7.9; median = 60) Gy. Because both groups were treated with similar doses of radiation, the following analyses were carried out with the groups of both 53 and 95 patients unless otherwise noted. To understand whether the survival of patients with lower doses radiation differed from that of those with higher doses, patients who received doses of the minimum to 25th percentile (QTR1) and those with 75th to the maximum dosage (QTR4) were compared using a Kaplan–Meier survival estimator. There was statistically no significant difference between the two groups (*p*-value = 0.57). Similarly, when patients with all stages were considered, there was no advantage of the higher-dose treatment over the lower-dose treatment or vice versa. These observations indicated that the variance in radiation dosage did not result in a significant difference in the survival outcome for this group of patients. Although cancers of other organs are also treated with IR frequently, OSCC cases provide a large volume of data for patients who have undergone radiation therapy, making them a valuable source of information for studying the resistance of tumors to radiation treatments.

### 3.2. Association of High Expression of APE1 and PRMT5 with Outcomes of Radiation Therapy

**Figure 1:**
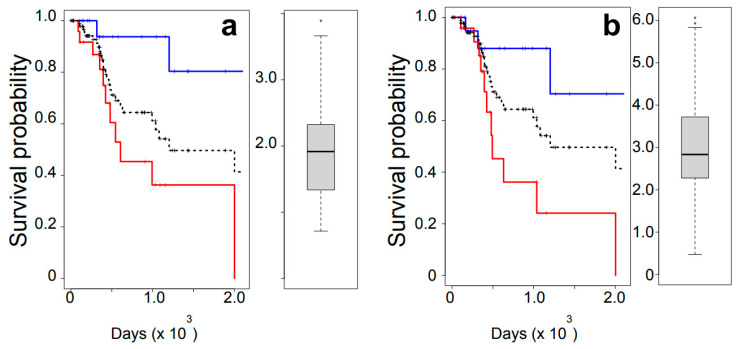
Poor outcomes associated with high expression of PRMT5 (**a**) and APE1 (**b**). Each left panel shows a Kaplan–Meier survival analysis for the lower (blue) and upper (red) quartile groups based on RNA expression (RNAseq). Survival curves including all patients are shown in dashed black lines. Differences in outcomes between the lower and upper quartile for APE1 and PRMT5: *p* < 0.05. Each right panel shows Tukey’s plot for the range of gene expression (RNA).

Using TCGA RNAseq and the survival records of OSCC patients, we elucidated whether the expression levels of PRMT5 and APE1 were associated with survival outcomes for OSCC patients who underwent radiation treatment. A total of 95 patients were grouped based on the expression levels of PRMT5 (Figure 1a) into QTR1 (the lower quartile; minimum to the first quartile) and QTR4 (the upper quartile; the third quartile to the maximum), and survival data were compared between these two groups. QTR4 patients (red line), i.e., those with tumor tissues expressing either PRMT5 or APE1 at higher levels, were associated with poorer outcomes compared to QTR1 patients (blue line) (Figure 1).

TCGA regularly profiles RNA expression in benign tissues in approximately 10% of the patients. We were interested in the expressional correlations in the normal oral mucosa between PPRMT5 and DSB repair genes found to be activated by PRMT5 [18] (Table 1). We evaluated the Pearson’s correlation coefficients of PRMT5 using RNAseq data from matched pairs of normal and carcinoma tissues (columns 2 and 3, Table 1). Statistically significant, positive correlations of PRMT5 expression were found with BRCA1, BRCA2, RAD51, RAD51AP1, XRCC5 (Ku86), and XRCC6 (Ku70). Interestingly, XRCC6 (Ku70), which was not described in the previous study, showed the highest correlation of expression with PRMT5 among the genes examined. Although only PRKDC (DNAPKcs) and XRCC6 (Ku70) showed high expressional correlation with PRMT5 among the matched tumor tissues (N = 43), high positive correlations of the PRMT5 levels were detected with BRCA1, PRKDC, RAD51, and XRCC6 when all the available tumor data were included (N = 522) (column 4, Table 1). Overall, the results were consistent with previous findings on the involvement of PRMT5 in DSB repair enhancement [18].

### 3.3. Tumor-Specific Co-Regulation of Genes with PRMT5

We extended the RNAseq correlation analysis to identify DNA repair genes that show high correlation with PRMT5. All the genes in the OSCC RNAseq database were evaluated to obtain Pearson’s correlation coefficients, and we thus found the genes that show a high positive correlation with PRMT5 (Table 2). The analysis identified APE1 (gene symbol = APEX1) as the protein whose expression is co-regulated by PRMT5 with the highest correlation coefficient among the approximately 15,000 genes analyzed, indicating that the expression levels of PRMT5 and APE1, two proteins involved in cellular IR recovery [18,19,20], were co-regulated. Therefore, we examined the dependency of the radiation survival on the level of APE1 expression (Figure 1b). As was the case for PRMT5, there was a statistically significant association between high expression of APE1 and poor outcomes of radiation therapy. 

**Figure 2:**
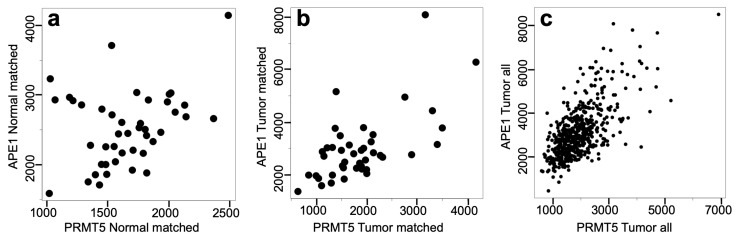
Tumor-specific co-regulation of PRMT5 and APE1. Expression of PRMT5 and APE1 in the normal (**a**) and tumor (**b**) tissues of matched cases, as well as in all the tumor tissues (**c**) were examined. Pearson’s correlation coefficient and their *p*-values: (**a**) 0.32; 0.038, (**b**) 0.644; 4.1 × 10^−6^, (**c**) 0.667; <2.2 × 10^−16^.

We analyzed the expression correlation between PRMT5 and APE1 in detail using a set of matched normal and the OSCC tissues (Figure 2). Unlike the high correlation observed with the OSCC tumor tissues, the correlation in the normal tissues was only marginal (Figure 2a). This result suggested that the co-regulation of PRMT5 and APE1 was caused by a mechanism specific to tumors.

### 3.4. Copy Number Variation as the Main Driver of the High Correlation of PRMT5-APE1 Expression

**Figure 3:**
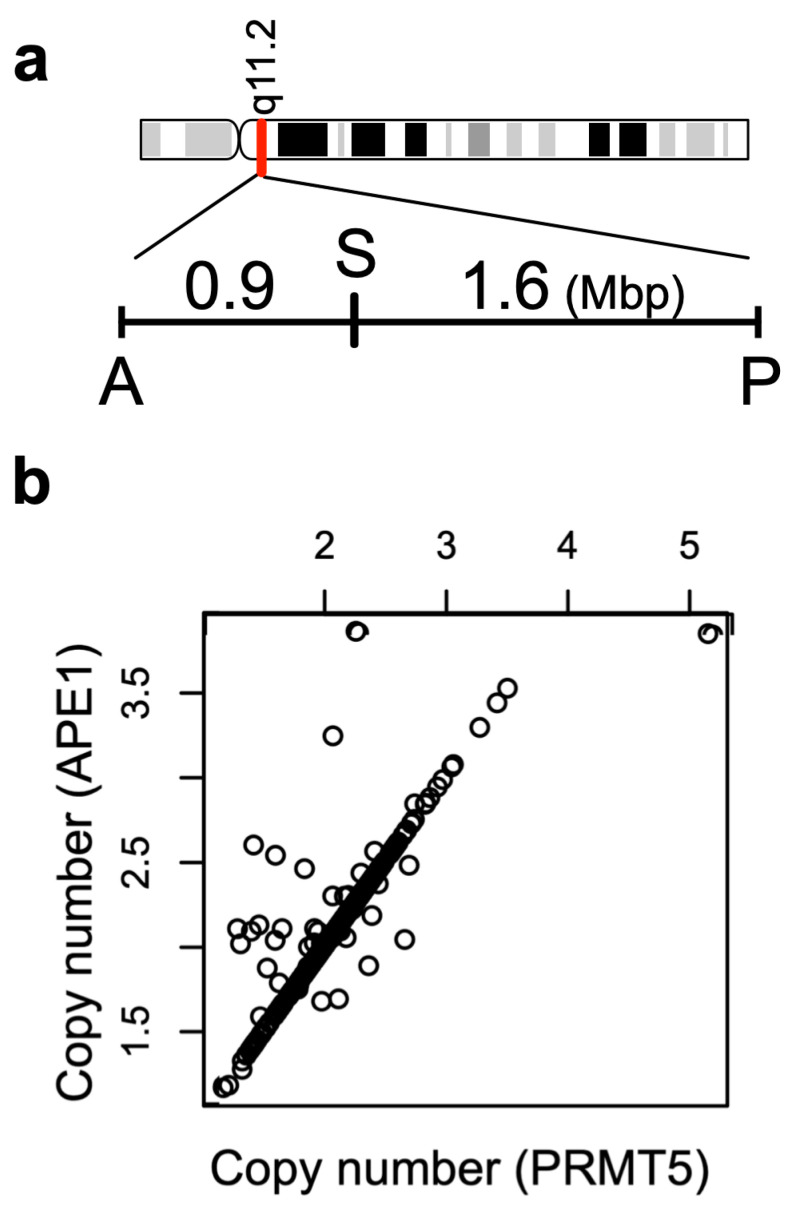
Near-identical CNV in APE1 and PRMT5 genes. (**a**) The coordinate of the APE1 and PRMT5 genes to show their close localization in the chromosome 14q11.2. A: APE1, P: PRMT5, S: SUPT16H. (**b**) Correlation plot of PRMT5 and APE1 CNV. N = 526. Pearson’s correlation coefficient = 0.88, *p* < 2.2 × 10^−16^.

**Figure 4:**
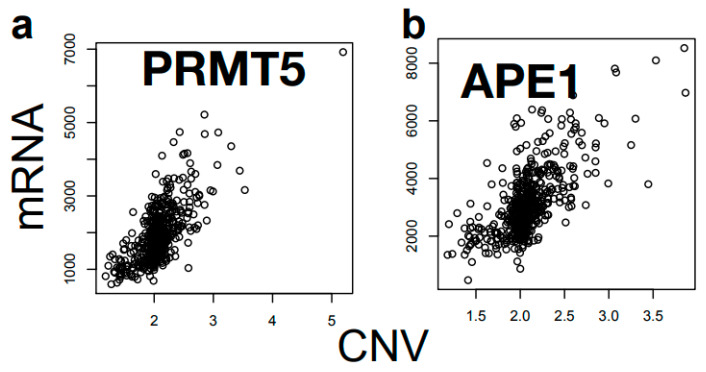
Plot of CNV vs. RNA levels for PRMT5 (**a**) and APE1 (**b**) genes showing high correlations, indicating that CNV becomes the major determinant of the expression levels of these genes in cancer cells.

Including APE1, the list of the genes that are co-regulated with PRMT5 is predominantly occupied by those localized in chromosome 14q11.2 in the genome (Table 2), suggesting a cis-acting regulatory mechanism. Because one of the most frequent genome alterations is copy number variations (CNVs), we compared the extent of CNVs precisely at the PRMT5 and APE1 gene locations, and found that the CNV between PRMT5 and APE1 genes was nearly identical (Figure 3). The distance between the transcription start sites of APE1 and PRMT5 genes is approximately 2.6 Mbp, based on the GRCh38 coordinate (Figure 3a). Thus, the near identical CNV between APE1 and PRMT5 is most likely due to the short distance between the two genes (Figure S1). There were strong correlations between the RNA expression level of PRMT5 and the CNV in the PRMT5 gene region (Figure 4). Likewise, this RNA–CNV correlation was also found in the case of APE1 (Figure 4). These observations led us to conclude that in cancer tissues, PRMT5 and APE1 are co-regulated, which reflects the extent of the CNV in the PRMT5-APE1 genome region.

### 3.5. Synergistic Sensitization of Cells to IR via Simultaneous Down-Regulation of PRMT5 and APE1

**Figure 5:**
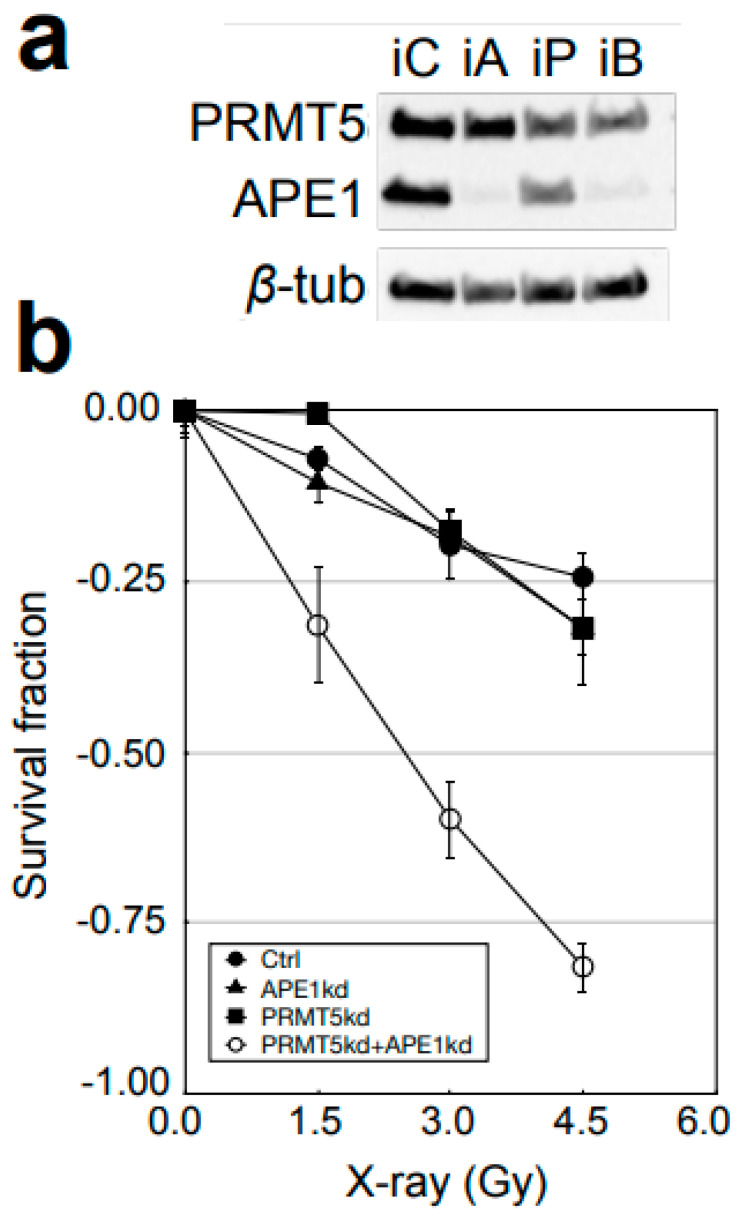
Cell survival against X-ray. (**a**) Immunoblot analysis of FaDu cells with PRMT5 and APE1 knockdown by siRNA. Extracts from FaDu cells with siRNA targeting none (control, iC), PRMT5 (iP), APE1 (iA), and both PRMT5 and APE1 (iB). (**b**) FaDu with down-regulation of (●) control siRNA (iC), (■) PRMT5 (iP), (▲) APE1 (iA), and (○) both PRMT5 and APE1 (iB) were irradiated and grown in colonies. *t*-tests (double-kd vs. PRMT5 kd) <0.01 in both survival assays at all the doses.

To elucidate the possible synergistic effect of PRMT5 and APE1 on cellular sensitivity to IR, we introduced siRNA to FaDu cells. Cells with stable siRNA knockdown (kd) were tested for their sensitivity to X-ray (Figure 5). FaDu derivatives with either PRMT5 or APE1 kd showed no significant difference in IR sensitivity. These results were different from previous studies that showed significant IR sensitizations in the single-gene kd of PRMT5 or APE1, but the difference was probably due to the use of different types of cancers and genetic backgrounds. When both PRMT5 and APE1 were down-regulated simultaneously, the cells became hyper sensitive to IR (Figure 5), indicating a synergistic cell sensitization via the PRMT5-APE1 double kd.

**Figure 6:**
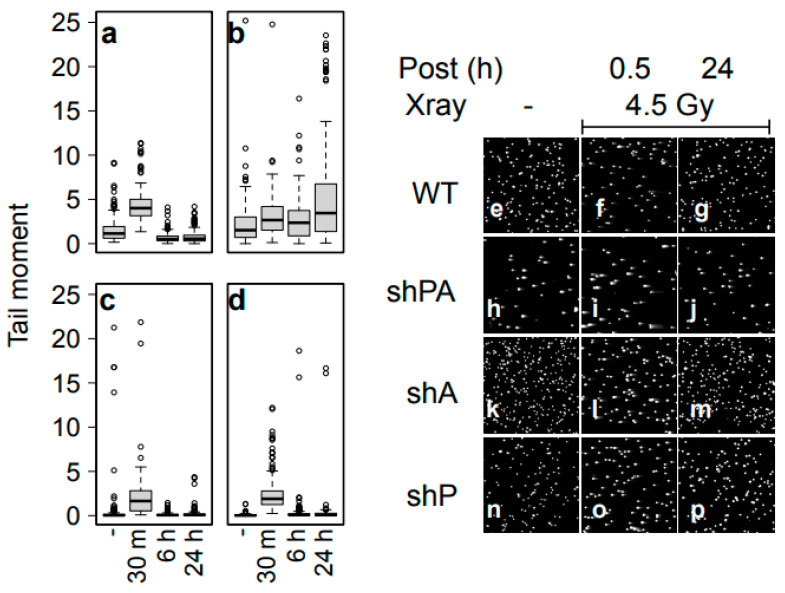
DNA damage and repair after exposure to X-ray. FaDu derivatives. ((**a**) control, (**b**) shAPE1 + shPRMT5, (**c**) shAPE1, and (**d**) shPRMT5) were irradiated at 4.5 Gy X-ray and incubated for the indicated time. The extents of DSBs were then analyzed with a comet assay in neutral pH conditions, and their tail moment values were calculated using CometAssay software (R&D system/Trevigen). (**e**–**p**) Fluorescent microscope images of the comet assay samples. (**e**–**g**) FaDu wild-type, (**h**–**j**) shAPE1 + shPRMT5, (**k**–**m**) shAPE1, (**o**,**p**) shPRMT5. (**e**,**h**,**k**,**n**) no X-ray, (**f**,**i**,**l**,**o**) 30 min after 4.5 Gy X-ray exposure, and (**g**,**j**,**m**,**p**) 24 h after 4.5 Gy X-ray.

To elucidate the effect of PRMT5 and APE1 kd on the efficiency of DSBR after X-ray radiation, single-cell gel electrophoresis (Comet assay) was carried out (Figure 6 and Table 3). The extent of DSB was assessed by carrying out a comet assay in neutral conditions, and assessment of the DNA tail moment after 0 or 4.5 Gy radiation followed by post-radiation incubation for 30 min, 6 h, and 24 h. In basal conditions (no radiation), double-kd cells (shPRMT5 + shAPE1) showed significantly more DSBs than the cells with single-gene kd (either the PRMT5 or APE1 kd). As predicted, after 30 min of IR treatment, all the cell lines showed increased DSBs. After 6 h post irradiation, while the amount of DSBs in the wild-type and the two single-kd cell lines decreased to levels that were even lower than the corresponding basal levels, the double-kd cells showed continually increased DSBs. At 24 h post irradiation, the damage was still significantly elevated in the double-kd cell lines, unlike the wild-type and the single-kd cell lines (Figure 6 and Table 3). These results suggested that the synergistic IR sensitization of the double-kd cells was caused both by the elevated basal level of the DNA damage stress and by the decreased DSB repair capacity of the cells. It should be noted that the basal level of the tail moment of the wild-type FaDu cells was higher than that of the single-knockdown derivatives, although the value was still lower than the basal level of the double-knockdown cells. The results were repeated at least three times and were reproducible. Therefore, the results implied that the basal level of DSB observed via the comet assay in the wild-type FaDu was higher than in the cells with APE1 or PRMT5 single knockdown. Currently, we do not have a clear mechanism to explain this observation, although it is possible that the results reflect the high cell growth (DNA replication) activity of the wild-type cells, leading to more DNA strand breaks. X-ray radiation not only activates DNA repair activities, but also signals cell-cycle arrest. After 6 and 24 h of repair time, the wild-type cells showed much fewer comet tails than the basal level, as the cell growth may not have started until the majority of DSBs had been repaired. We do not have a clear mechanism explaining why single-knockdown cells exhibited fewer comet tails than the wild-type at the basal level, but it is plausible that cell growth activities were slightly slowed down by the single knockdowns. In any case, it is evident that the simultaneous knockdown of PRMT5 and APE1 made the cells unable to repair in a timely manner, and caused an increased damage burden after 24 h, unlike the wild-type and both the single-knockdown cells.

### 3.6. Synergistic Sensitization of Tumors in Xenograft

**Figure 7:**
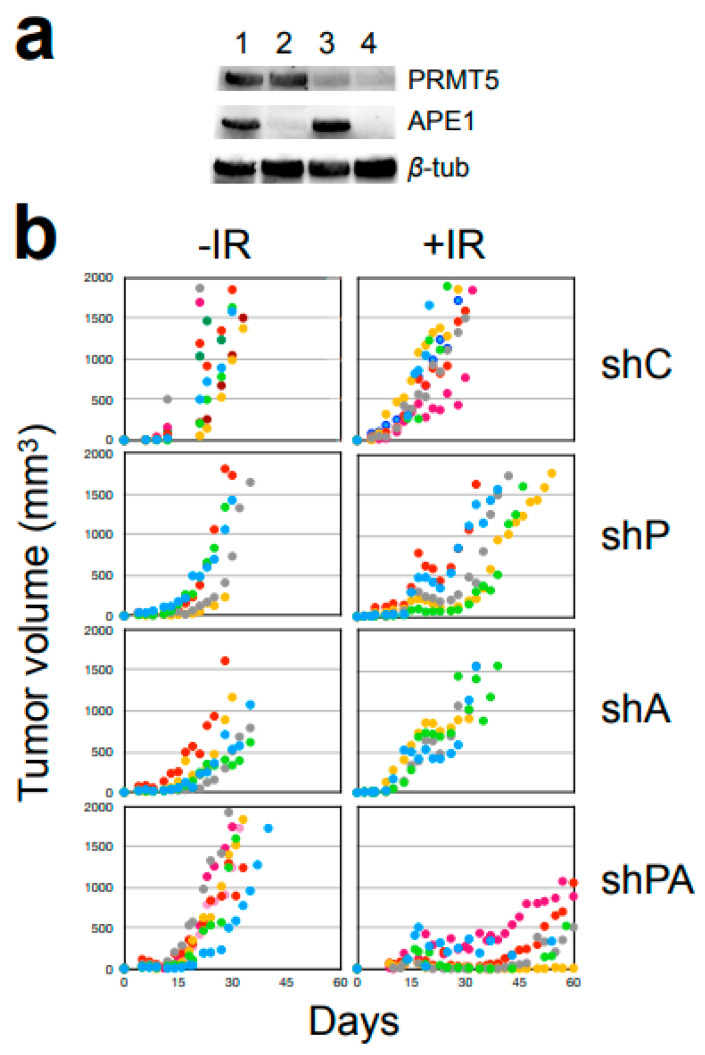
Tumor growth post X-ray radiation in a FaDu xenograft. (**a**) FaDu derivatives. 1: sh-control (shC), 2: sh-APE1 (shA), 3: sh-PRMT5 (shP), 4: sh- PRMT5+APE1 (shPA or shB). (**b**) Athymic mice injected with FaDu with shRNA kd were exposed with 12 Gy (4 Gy × 3 day) at the tumor sites locally in PXi 225XL-RAD, and the growth of tumor size was measured twice a week with the formula 6/π • (wider length) • (shorter length) 2.

To test the effect of the PRMT5-APE1 down-regulation in vivo, a stable down-regulation of the PRMT5 and APE1 was necessary for tumor growth. Therefore, stable derivatives of FaDu cells harboring the control, shRNA for PRMT5 down-regulation, and shRNA for APE1 down-regulation (shCtrl, shPRMT5, shAPE1, and shPRMT5+shAPE1) were generated. The FaDu derivatives were transplanted into athymic mice, and the effectiveness of fractionated X-ray irradiation (3 × 4 Gy, total 12 Gy) in controlling tumor growth was examined (Figure 7) via a longitudinal tumor growth comparison using the R TumGrowth statistical software package [35]. There was no significant difference in the tumor growth without IR intervention among the four FaDu derivatives (Figure 7b and Table 4), indicating that the downregulation of PRMT5 and/or APE1 did not affect tumor growth. After X-ray irradiation, the tumor growths were slowed but eventually regrew, and no statistical significance was found in the longitudinal analysis between the control and the single-kd cells (Table 4). However, there was a significant growth delay in the double-kd cells compared to the control (Table 4). Thus, PRMT5 and APE1 down-regulation synergistically sensitized the tumors to the radiation treatment.

### 3.7. CNV at the APE1-PRMT5 Gene Region in OSCC Tumors

Using a NanoString CNV panel with a code set specific to the APE1-PRMT5 gene region, we determined CNV values using genomic DNA extracted from formalin-fixed, paraffin-embedded OSCC tissues (Figure S2). The NanoString signal intensities were normalized using the values for genomic DNA from a human diploid control specimen (Promega). Focusing on the code set of the genome region that includes nine genes including APE1 and PRMT5 genes, we found a statistically significant copy number increase in the APE1 and PRMT5 genes (Figure S2 and Table S1). This observation was consistent with the reports that PRMT5 and APE1 expressions are increased in tumor tissues compared to normal tissues [23,24].

## 4. Discussion

IR generates clusters of DNA lesions composed of DSB and oxidative damage [12,36]. While DSBs generated directly by IR pose an immediate risk to cells, oxidative damage causes DNA breaks as well; thus, cells need to repair both types of damage to survive. PRMT5 and APE1 enhance the repair of DSB and oxidative DNA damage, respectively, and thus are critical for cellular recovery from IR-induced DNA damage.

Genes highly expressed in cancer cells are often associated with aggressive cell growth and survival. PRMT5 and APE1 are among these genes [20,24], at least partly because of enhanced DNA repair for DSB and oxidative DNA damage, respectively. Tumors that express both PRMT5 and APE1 highly may therefore gain further resistance to insults that generate both types of DNA damage, including IR. The present study elucidated an expressional correlation between PRMT5 and APE1 for the first time.

**Figure 8:**
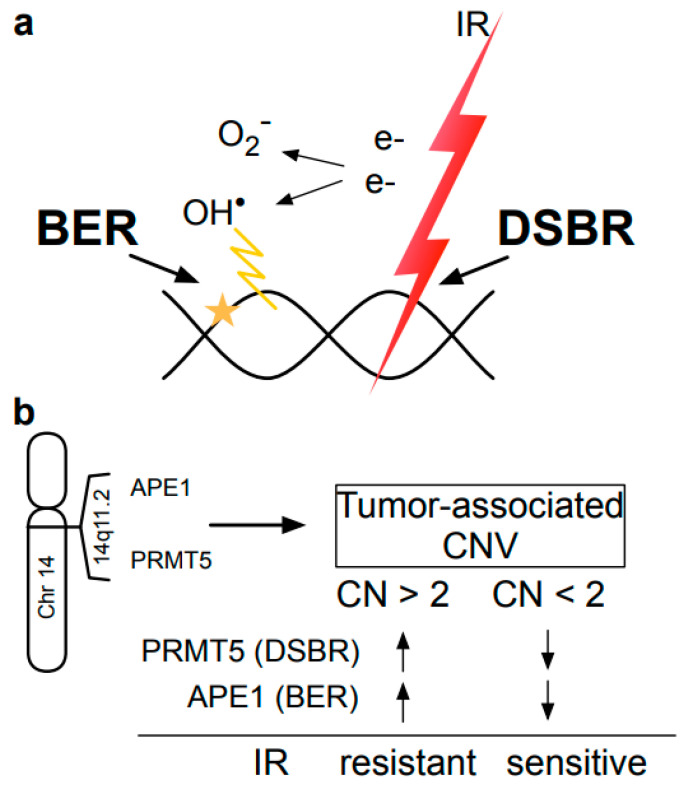
A model associating acquired IR resistance of tumors with CNV in 14q11.2. (**a**) IR-generated clustered DNA lesions containing DSBs, SSBs, and oxidized base damage require both DSBR and BER to survive. (**b**) PRMT5 and APE1 genes localized in a 2.5 Mbp genome region of 14q11.2 share almost identical CNVs in the tumor tissues, and exhibit transcriptional co-regulation. In the 2.5 Mbp, 14q11.2 region contains not only PRMT5 and APE1 but also SUPT16H, PARP2, METTL3, and other genes that have been associated with crucial DNA repair and DNA damage response activities. The model predicts that tumors with high copy numbers in the PRMT5-APE1 genome region are associated with acquired IR resistance, and those with low copy numbers may be sensitized due to their lower levels of APE1 and PRMT5.

Based on TCGA RNAseq data, a high expressional correlation between PRMT5 and APE1 was found in OSCC tumor tissues. Such high expressional correlation was not observed with the matched normal tissues. Seeking a molecular mechanism that explains the tumor-specific high correlation between PRMT5 and APE1 expression, we consider it significant that the genomic locations of PRMT5 and APE1 are very close to each other at chr 14q11.2 (Figure 8).

CNV frequently occurs in the cancer genome, and the chance of sharing an equal CNV value in a cell is higher between two genes that are closely localized in a chromosome. The extent of CNV was almost identical between the PRMT5 and APE1 genes. Importantly, there were high correlations between RNAseq and CNV values for both APE1 and PRMT5. These observations suggest that the extent of CNV in the APE1-PRMT5 genome region is a critical factor in the co-regulation of APE1-PRMT5. DNA replication stress due to uncontrolled cell growth and/or DNA damage via therapeutic procedures are expected to increase genome instability, which may be the cause of increased CNVs in cancer cells. Our assessment of TCGA data and our CNV analysis of FFPE tissues indicate that sub-populations of cells in tumor tissues contain copy number increases in the APE1-PRMT5 region. We speculate that these cells gain growth and survival advantage against IR treatment to affect the outcome of the radiotherapy significantly, as high expression levels of PRMT5 and APE1 have been associated with poor outcomes of radiation therapy in oral cancer patients. Previous studies are consistent with this prediction that high expression of APE1 in OSCC and glioblastoma were linked to tumor resistance to alkylating reagents, reactive oxygen species [37,38], and chemoradiotherapy [39,40,41]. Indeed, in this study, cells were synergistically sensitized to IR via the simultaneous down-regulation of PRMT5 and APE1. There was a significant increase in DSBs in the double-kd cells even without radiation, suggesting that there was an increase in the basal number of DSBs in the cells. These observations suggest that the expressional co-regulation of PRMT5 and APE1 significantly impact cellular resistance to IR (Figure 8).

The chromosomal locus contains other genes that have been associated with DNA repair (Table 2). Among them, SUPT16H, a subunit of a histone chaperone FACT, has been reported for its crucial role in DSB repair [42]. Similar to PRMT5 and APE1, the downregulation of SUPT16H significantly sensitized a breast cancer cell line MCF7 to IR [42]. PARP2, which has recently been shown to bind to DSBs with histone parylation factor 1 (HPF1), presumably to facilitate DSB rejoining [43], is also in the region; its expression is highly correlated with that of PRMT5 and APE1. Other genes in the cluster have also been reported for their involvement in DNA damage responses. TOX4 (TOX HMG Box family member 4) has been reported to bind cisplatin adducts in DNA [44]. METTL3 has been intensely studied for its crucial roles in DNA damage response via the regulation of RNA methylation at N6-methyladenosine (m^6^A) [45]. Therefore, it is possible that an increase in the copy number in the PRMT5-APE1 genome region provides multiple layers of protection against radiation and other DNA-damaging reagents. More studies are necessary to determine the contribution of genes other than PRMT5 and APE1 to resistance to radiation and chemotherapeutic drugs including cisplatin.

Finally, it should be noted that CNV only partially explains the mechanism of the co-regulation of the PRMT5 and APE1 genes, because it does not explain expression patterns significantly different from those of the normal (diploid) cells. We speculate that the loss of normal gene regulation, presumably due to epigenetic alterations during tumorigenesis, results in increased reliance on gene dosage (CNV) alone. Establishing this mechanism may open up opportunities to simultaneously regulate these DNA repair factors in the genome region, which should improve therapeutic strategies for patients who may otherwise have a poor prognosis of radiation treatment due to an increase in copy number in the PRMT5-APE1 locus.

## Figures and Tables

**Table 1:** Correlation of gene expression of PRMT5 with DSBR genes.

Gene	Correlation Coefficient (vs. PRMT5)		
	Matched (N = 43)		All Tumors (N = 522)
	Normal	Tumor	Tumor
PRMT5	1	1	1
BRCA1	0.367 *	0.18	0.124 *
BRCA2	0.306 *	0.265	−0.024
NHEJ1	−0.057	−0.037	0.098 *
PRKDC	0.289	0.4 *	0.147 **
RAD51AP1	0.566 **	0.251	0.115 **
RAD51	0.708 **	0.239	0.17 **
XRCC4	0.446 **	0.112	0.045
XRCC5	0.625 **	−0.096	0.01
XRCC6	0.726 **	0.348 *	0.274 **

* *p* < 0.05; ** *p* < 0.01.

**Table 2:** Genes co-regulated with PRMT5. All genes in the OSCC RNAseq database were analyzed for the correlations of their expression with that of PRMT5. r: Pearson’s correlation coefficient; *p*: *p*-values adjusted using Bonferroni correction; cytoband: information from DAVID (https://david.ncifcrf.gov, accessed on 2 September 2023). Genes linked to DNA repair activities are in bold. The entire list is shown in Table S1. * and **: Pearson’s correlation coefficient tests. * *p* < 0.01, ** *p* < 0.001.

Gene Symbol	r	*p*	Cytoband
APEX1	0.667	2.07 × 10^−64^	14q11.2
NGDN	0.664	1.93 × 10^−63^	14q11.2
IPO4	0.663	2.49 × 10^−63^	14q12
OXA1L	0.618	4.43 × 10^−52^	14q11.2
SUPT16H	0.615	1.71 × 10^−51^	14q11.2
C14orf119	0.612	1.14 × 10^−50^	14q11.2
PSMB5	0.601	2.62 × 10^−48^	14q11.2
GMPR2	0.538	2.85 × 10^−36^	14q12
PARP2	0.530	5.60 × 10^−35^	14q11.2
METTL3	0.529	8.76 × 10^−35^	14q11.1
TOX4	0.527	1.64 × 10^−34^	14q11.2
AP4S1	0.527	3.34 × 10^−32^	14q12

**Table 3:** Comet assay evaluation. Cells that were unirradiated and irradiated (4.5 Gy, 24 h-incubation) were analyzed using their tail moment values, which were determined with CometAssay software. *p*-values (Welch’s *t*-test) at 24 h of wild-type APE1 knockdown and PRMT5 knockdown compared to double knockdown are shown with Bonferroni’s p adjustment.

	X-ray	Post Incubation	Number of Cells Analyzed	Tail Moment	
				Mean ± sd	*t*-Test (vs. shBoth)
WT	-	-	277	1.46 ± 1.29	
	4.5 Gy	24 h	152	0.77 ± 0.74	<0.001
shBoth	-	-	161	2.23 ± 2.64	
	4.5 Gy	24 h	125	5.62 ± 6.25	-
shAPE1	-	-	165	0.58 ± 2.70	
	4.5 Gy	24 h	106	0.27 ± 0.69	<0.001
shPRMT5	-	-	89	0.09 ± 0.21	
	4.5 Gy	24 h	101	0.49 ± 2.28	<0.001

**Table 4:** Tumor growths among groups (Figure 7) were compared based on a linear mixed model with Bonferroni adjustment using the R-package TumGrowth.

X-ray	Pairs	*p*-Value
-	WT vs. shPRMT5	1
	WT vs. shAPE1	0.247
	WT vs. shBoth	1
+	WT vs. shPRMT5	0.151
	WT vs. shAPE1	0.762
	WT vs. shBoth	<0.0001

## Data Availability

Data in this report will be shared upon request.

## Supplementary Materials

The following supporting information can be downloaded at: https://www.mdpi.com/article/10.3390/cells12202425/s1, Figure S1: Evaluation of CNV in chromosome 14. Figure S2: CNV in the chr14 APEX1-PRMT5 genome region of oral cancer tissues. Table S1: Mean CNV in the indicated genes was evaluated based on Nanostring data from eleven oral cancer tissues.
