# Supplementary material for: Copy Number Variation That Influences the Ionizing Radiation Sensitivity of Oral Squamous Cell Carcinoma
Source: Cells. 2023 Oct 10;12(20):2425. doi: 10.3390/cells12202425 (PMC10605269; doi:10.3390/cells12202425)
Supplement: Supplementary file 1 [file cells-12-02425-s001.zip › cells-2621078-supplementary.pdf]

Table S1, Izumi et al.

Mean CNV at indicated genes were evaluated based onthe Nanostring data on 11 the oral cancer tissues. sd: standarddeviation. Welch's t.test was performed comparing the CNV of oralcancer tissues to that of control human diploid DNA (Promega). * $p < 0.05$.

Table S1. Gene-specific CNV with a Nanostring custom panel

	PARP2	APEX1	HNRNPC	SUPT16H	TOX4	METTL3	PRMT5	NEDD8	GMPR2
mean	1.036	1.095	1.338	0.941	1.003	1.124	1.232	1.502	1.192
sd	0.088	0.103	0.165	0.134	0.117	0.097	0.184	0.353	0.088
p*	0.5291	0.0476	4.00E-04	0.5291	0.9332	0.0104	0.0104	0.0057	3.00E-04

Figure S1, Izumi et al.

Evaluation of CNV in chr 14 (Top) The method to determine CNV. SNP array for the oral squamous cell carcinoma data in TCGA was converted to the copy number. SNP probes corresponding to Chr 14 were screened at 100 kb interval, and intensities of identified SNP probes were converted to copy number values. The set of CNV values were determined for all the OSCC tissues (N = 526). (Bottom) Correlation coefficients to the CNV at the PRMT5 gene location were determined with CNVs at all the locations, and blotted along the chr14 location.

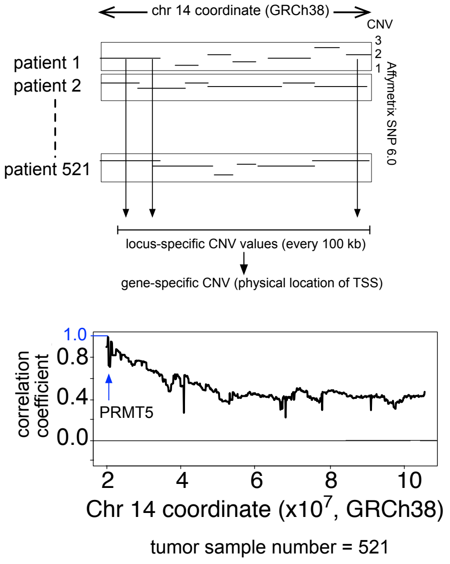

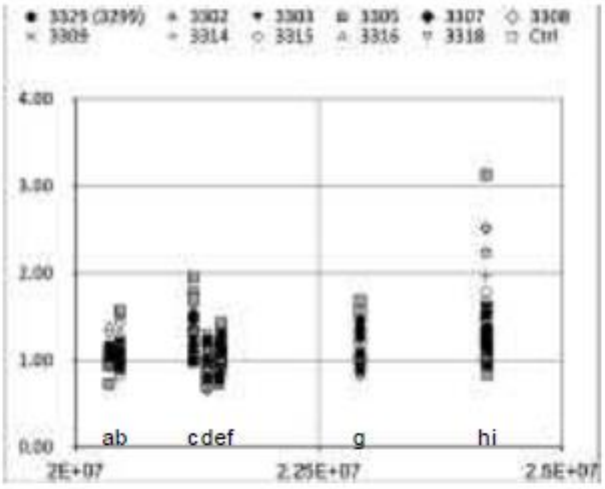

Figure S2. CNV in the chr14 APEX1-PRMT5 genome region of oral cancer tissues. Genome DNA extracted from 11 FFPE oral cancer tissues and a normal human genome DNA were analyzed with a NanoString panel that include genes in the region. a: PARP2, b: APEX1, c: HNRNPC, d: SUPT16H, e: TOX4, f: METTL3, g: PRMT5, h: NEDD8, i: GMPR2. Three probes per gene were used in the panel.
